# Fabrication and characterization of polycaprolactone/chitosan nanofibers containing antibacterial agents of curcumin and ZnO nanoparticles for use as wound dressing

**DOI:** 10.3389/fbioe.2022.1027351

**Published:** 2022-09-23

**Authors:** Pezhman Mosallanezhad, Hossein Nazockdast, Zahed Ahmadi, Amir Rostami

**Affiliations:** ^1^ Department of Polymer Engineering, Amirkabir University of Technology, Mahshahr, Iran; ^2^ Department of Polymer Engineering and Color Technology, Amirkabir University of Technology, Tehran, Iran; ^3^ Department of Chemistry, Amirkabir University of Technology, Tehran, Iran; ^4^ Department of Chemical Engineering, Faculty of Petroleum, Gas, and Petrochemical Engineering, Persian Gulf University, Bushehr, Iran

**Keywords:** polycaprolactone (PCL), chitosan, zinc oxide, curcumin, wound dressing, drug release, antibacterial activity

## Abstract

The potential of the nanoscale structure is utilized by electrospun nanofibers, which are promising materials for wound dressings. Here, we prepared wound dressings constituting polycaprolactone (PCL) and chitosan (CS). Curcumin (Cur) and zinc oxide nanoparticles (ZnO) as antibacterial agents were embedded in PCL/CS electrospun nanofibers and different properties including morphology, physicomechanical, interaction with water, antibacterial efficiency, and *in vitro* studies were investigated. SEM images confirmed the nanofibrous structure of samples with 100 ± 5 to 212 ± 25 nm in average diameter. Elemental analysis of nanofibers showed a good distribution of ZnO along nanofibers which not only caused decreasing in nanofiber diameter but also increased tensile strength of nanofibers up to 2.9 ± 0.5 MPa and with good elongation at break of 39 ± 2.9. ZnO nanoparticles also facilitated the interaction of nanofibers with water, and this led to the highest water vapor transition rate, which was equal to 0.28 ± 0.02 g cm^−2^ day^−1^. The sample containing 3 wt% Cur had the highest water uptake value (367 ± 15%) and the lowest water contact angle (78 ± 3.7°), although Cur has a hydrophobic nature. The release profile of Cur showed a two-stage release and the Peppas model predicted a non-fickian diffusion. Simultaneous incorporation of CS, ZnO, and Cur effectively inhibited bacterial growth. In addition, *in vitro* studies represented that high content of Cur decreases cell viability and cell attachment. The outcomes from the fabricated nanofibrous scaffolds demonstrated appropriate properties for application as a wound dressing.

## Introduction

In light of the advancements made in tissue engineering, the topic of electrospinning is gaining popularity in the biomedical world. The high available surface area and high porosity of electrospun nanofibers, being affordable and easy to handle, make electrospun nanofibers an ideal option for wound dressing (Kakoria and Sinha-Ray, 2018; Huang et al., 2022). Electrospun nanofibers can be formed by a range of natural polymers such as cellulose, chitosan (CS), collagen, gelatin, and silk, as well as synthetic biodegradable polymers such as polylactic acid (PLA), polyglycolic acid (PGA), and polycaprolactone (PCL) (Mendes et al., 2017; Zhang et al., 2022).

Electrospinning of pure CS is accompanied by several challenges associated with the limited solubility of pure CS in most organic solvents, its typically large molecular weights, and strong physical networks from hydrogen bonds (Pillai and Sharma, 2009; Qasim et al., 2018). To address this issue, the blending of CS with other natural and synthetic polymers such as polyethylene oxide (PEO), polyvinyl alcohol (PVA), PLA, and collagen have been reported so far. In fact, these polymers, which are well-known for their fiber-forming ability, were used to facilitate the electrospinning of CS (Jia et al., 2007; Chen et al., 2008; Lou et al., 2008; Xu et al., 2009). PCL is an aliphatic semi-crystalline biodegradable polyester known for its good mechanical properties. Being non-toxic and tissue compatible, it has been used in different biomedical applications such as tissue engineering scaffolds, resorbable sutures, and drug delivery (Janmohammadi and Nourbakhsh, 2019; Dwivedi et al., 2020). Nanofibers from pure PCL and its blends with other biopolymers like PLA, gelatin, and polyethylene glycol (PEG) were also produced by many researchers through the electrospinning method (De Prá et al., 2017; Radisavljevic et al., 2018; Janmohammadi and Nourbakhsh, 2019; Semitela et al., 2020).

In recent years, electrospun PCL/CS has received a great deal of attention for the preparation of nanofiber scaffolds (Saatcioglu et al., 2021). PCL/CS nanofibers loaded with tetracycline hydrochloride (TCH) (Ghazalian et al., 2022), azithromycin (Alimohammadi et al., 2022), and metformin (Zhu et al., 2020) and its wound dressing containing aloe vera (Yin and Xu, 2020), Melilotus officinalis (Shahrousvand et al., 2021), and Jaft (Hashemi et al., 2021) have been recently reported in the literature. For instance, Ghazalian et al. (2020) prepared TCH-loaded PCL/CS core-shell nanofibrous structure. The prepared samples demonstrated a two-stage behavior of drug release, with an initial burst release stage followed by a sustained release stage. In another study by Alimohammadi et al. (2020), PCL/CS nanofibers containing azithromycin were prepared *via* electrospinning which showed a sustained release of the drug with minimized burst release. Zhu et al. (2020) also prepared crosslinked PCL/CS nanofibrous scaffolds as a carrier for the metformin drug. They showed that crosslinked samples were more suitable for drug release, osteogenic differentiation, cell adhesion, and bone mesenchymal stem cells.

In addition, with the development of electrospun nanocomposite scaffolds containing nickel nanoparticles (Karakas et al., 2017), graphene oxide (Aidun et al., 2019), hydroxyapatite (Shirzaei Sani et al., 2021), and multi-walled carbon nanotubes (Mirmusavi et al., 2022) enhanced functional performances of complex nanostructures have been demonstrated. For example, Aidun et al. (2019) investigated the biological activity and physicochemical properties of the PCL/CS scaffolds incorporated with graphene oxide. They found that an increase in nanoparticles corresponded to an increase in the hydrophilicity, bioactivity, cell adhesion, and proliferation of the scaffolds. Sani et al. (Shirzaei Sani et al., 2021) incorporated hydroxyapatite nanoparticles onto PCL/CS nanofibers to enhance their mechanical properties, proliferation, cell viability, and bioactivity of them. In another work, the structural, mechanical, and biological properties of PCL/CS electrospun nanocomposite scaffolds containing multi-walled carbon nanotubes were studied by Mirmusavi et al. (2022). The samples containing nanoparticles showed more cell viability and bioactivity compared to the neat samples. Among these investigations, no research on the Cur drug and ZnO nanoparticles has been seen. Cur is a natural polyphenol with antioxidant, anti-tumor, and anti-inflammatory properties (Afshar et al., 2019; Al-Bishari et al., 2022). The US Food and Drug Administration (FDA) has designated Cur as Generally Recognized As Safe (GRAS) and clinical trials have demonstrated good tolerability and safety profiles even at doses up to 12,000 mg/day of a 95% concentration (Hewlings and Kalman, 2017). Furthermore, ZnO is a multifunctional nanoparticle with effective antimicrobial and photocatalytic properties, biocompatibility as well as being nontoxic and inexpensive. ZnO can also promote the scaffold surface’s hydrophilicity resulting in cell attachment and proliferation (Karthikeyan et al., 2020). It is well known that curcumin can chelate zinc metal ions strongly by acting as a ligand and forming stable complexes. The use of this substance may represent a novel strategy for the early detection and treatment of chronic illnesses (Prasad et al., 2021).

In this study, we electrospun a homogeneous mixture of CS and PCL and evaluated the success of creating nanofibrous scaffolds with the desired morphology. For the first time, Cur and ZnO were simultaneously added to the PCL/CS nanofibrous scaffolds because the antibacterial activity of CS-containing nanofibers is limited by the NH2 groups of the CS backbone (Korniienko et al., 2022). The findings of the investigation into the structural, physicomechanical, and biological characteristics of the prepared scaffolds demonstrated their appropriate properties as wound dressings.

## Experimental

### Materials

PCL granules with average molecular weight (Mn) of 80,000 g mol^−1^, and density of 1.145 g cm^3^, CS with medium molecular weight and degree of deacetylation of 80%–85% (CAS Number: 9012-76-4), Cur drug, Hexafluoroisopropanol (HFIP), 3- [4,5- dimethylthiazol-2-yl]-2,5 diphenyltetrazolium bromide (MTT), Phosphate-buffered saline (PBS), methanol, and ethanol were purchased from Sigma-Aldrich (Germany). ZnO with particles size of 10–30 nm, specific surface area of 20–60 m^2^ g^−1^ and purity of 99+% was purchased from US Research Nanomaterials, Inc (United States). The *Staphylococcus aureus* (*S. aureus*, ATCC 25,923), *Escherichia coli* (*E. coli*, ATCC 25,922), and Fibroblast cells (L929) were purchased from the Pasteur Institute of Tehran (Iran).

### Scaffolds fabrication method

To determine the optimum condition of electrospinning a full factorial design experiment was conducted. Additional information is provided in the Supplementary Material (Supplementary Table S1). In brief, it was found that the optimal PCL concentration, CS concentration, flow rate, and voltage were 15 wt%, 3 wt%, 15 kV, and 1 ml h^−1^, respectively. The needle-to-collector distance and collector speed were fixed at 15 cm and 300 rpm, respectively. To prepare the electrospinning solution, a 15 wt% PCL solution in HFIP was first prepared by stirring (500 rpm) at ambient temperature for 120 min. Then CS was gradually added to the previous solution to reach a total concentration of 3 wt% and stirred (500 rpm) for 90 min. In the case of samples containing Cur and ZnO, they were added to the PCL/CS solution and sonicated for 30 min before electrospinning. A transition from yellow to orange was seen after the sonication process was finished. Finally, the prepared solutions were loaded in a 5 ml syringe with a standard blunt end needle (20 G) in the electrospinning setup. The electrospun nanofibers were placed in an oven for 24 h to dry completely. The sample codes and concentrations are listed in Table 1.

**TABLE 1:** Sample codes and their corresponding compositions.

Concentration (wt%)				Sample code
PCL	CS	ZnO	Cur	
15	0	0	0	PCL15
15	3	0	0	PCL15CS3
15	3	1	0	PCL15CS3ZnO1
15	3	1	1	PCL15CS3ZnO1Cur1
15	3	1	3	PCL15CS3ZnO1Cur3

### Characterization method

The chemical structure of samples was investigated by attenuated total reflectance Fourier transform spectroscopy (ATR-FTIR). The spectrum was recorded through 64 scans at a resolution of 4 cm^−1^ and in the range of 600–4000 cm^−1^ by FTIR spectrometer of Thermo Nicolet Nexus 670 (Madison, WI, United States).

The morphology of scaffold surfaces was evaluated with a scanning electron microscope (SEM, AIS 2100, Seron Technology, South Korea) with an acceleration voltage of 20 kV. The distribution of ZnO nanoparticles incorporated into samples was investigated by using a scanning electron microscope with energy dispersive X-ray spectroscopy (TESCAN. MIRA II, France) with an accelerating voltage of 10 kV.

The physical state of ZnO incorporated in samples was analyzed with X-ray diffraction (XRD, EQuniox 3000, Inel, France) in the 2θ range of 5–80°. Applied radiation was Cu Kα with 40 kV, λ = 0.15418 nm, and 30 mA at room temperature.

To investigate the mechanical properties, 5 replicates of each sample (50 mm × 10 mm × 2 mm) were stretched at a rate of 5 mm min^−1^ until the fracture point according to ASTM D882 on a universal material testing machine (Instron 5566, United States).

Thermogravimetric analysis was used to assess the thermal behavior of the materials (TGA, Q-500, TA Instruments, United States). Under a nitrogen atmosphere, the pre-weighed samples (15 mg) were heated at a rate of 10°C.min^−1^ from 25 to 550°C.

The hydrophilicity of the samples was evaluated using the sessile drop method by contact angle goniometer (OCA20, Data Physics Instruments, Germany). To this end, a drop of distilled water was placed on the surface of samples (10 × 10 mm^2^) using a 0.3 ml syringe. The contact angle of water drop on the surface of the samples was recorded after a few seconds from both sides of the drop. The reported contact angle was the mean of 5 replicates at 5 different sites of a sample.

To evaluate the water uptake behavior of the scaffolds, the samples were accurately weighed and then immersed in distilled water at 37°C. After immersion, the samples were weighed at regular intervals. The percent of water uptake in the samples was calculated based on the Eq. 1 (Ghaee et al., 2019):

$$Water\;Uptake\;\left(\%\right)=\frac{W_{w}-W_{d}}{W_{d}}\times 100$$ (1)

Where W_d_ and W_w_ represent the weight of dry and submerged samples, respectively.

To investigate the ability to control water loss, the water vapor transmission rate (WVTR) of samples was analyzed according to ASTM E96/E96M-10. The samples covered the opening of the 5 ml glass vial containing 1 ml distilled water. The container was placed in the humidity chamber with a constant temperature of 37°C and humidity of 25% for 48 h. A similar container without any sample was also placed in the humidity chamber as a control. WVTR was calculated using the Eq. 2 (Fahimirad et al., 2021):

$$WVTR\;\left(g.{cm}^{-2}.{day}^{-1}\right)=-\frac{\Delta W}{A\times \Delta t}$$ (2)

Where ΔW is the variation of weight (g) of water before and after the test, A is the exposure area of the samples (cm^2^), and Δt is the test time (day).

To investigate the *in vitro* degradation behavior of samples under physiological conditions, the samples (20 × 20 mm^2^) were immersed in PBS (pH = 7.4) and incubated at 37°C. The PBS solution was refreshed every 48 h. At each time point, the sample was removed from the solution and dried at 50°C. the weight loss of samples was calculated based on the Eq. 3 (Varshosaz et al., 2021):

$$Weight\;loss\left(\%\right)=\frac{W_{i}-W_{t}}{W_{i}}\times 100$$ (3)

Where W_i_ and W_t_ are the initial dry weight and the final dry weight of the samples (n = 3).

To investigate the release profile of Cur drug, samples (1 × 1 cm^2^) were immersed in 5 ml of PBS (pH = 7.4) and incubated at 37°C. At specific time points, 1 ml of the media was extracted and replaced with 1 ml of fresh media. To determine the concentration of the sample, 5 solutions of Cur were prepared with different concentrations, and their maximum absorbance at 425 nm was measured by UV-Vis (Secomam, France).

The Eq. 4 was used to fit Peppas’ model to the experimental data to study the kinetics of Cur release from electrospun nanofibers (Lao et al., 2008):

$$\frac{M_{t}}{M_{\infty }}=Kt^{n}$$ (4)

Where *K* and *t* are the kinetic constant and release exponent, and *M*_*t*_ and *M*_*∞*_ are the amount of drug released at time *t* and the total amount of drug, respectively.

The antibacterial activity of samples was investigated using two types of bacteria, a Gram-positive (*S. aureus*) and a Gram-negative (*E. coli*) according to agar diffusion method at 37°C. The strip-shaped samples (20 × 20 mm^2^) were immersed in methanol (90%) and PBS (pH = 7.4) for 15 min to neutralize and then sterilized by Gama radiation with a dose of 15 kGy. The samples were then suspended in bacterium solution and incubated in at 37°C in a shaking incubator for 24 h. The control was a suspension culture without any samples. The optical density of the samples was read by spectrophotometer at wavelength of 600 nm. The antibacterial efficiency was calculated based on the Eq. 5 (Chen et al., 2017):

$$Antibacterial\;efficiency\;\left(\%\right)=\left(1-\frac{{OD}_{1}}{{OD}_{2}}\right)\times 100$$ (5)

Where OD_1_ and OD_2_ are the optical densities of the control medium and the bacteria in the solution-containing sample, respectively.

Cell viability of samples was investigated by MTT assay in direct mode. First, samples were sterilized using Gama radiation with a dose of 15 kGy. Suspensions of L929 cell line were seeded on the sterilized samples with a density of 5 × 10^4^ cells per well and stored in an incubator (at 37°C, 5% CO_2_, and 90% humidity). After 24 h, the medium of each well was replaced with 200 μL of 0.5% MTT solution and incubated for 4 h. The MTT solution of each well was replaced with 150 µL isopropanol and the absorbance was read by ELISA at wavelengths of 490 and 630 nm as reference. Cell viability of samples was calculated by the Eq. 6 (Ghaee et al., 2019):

$$Cell\;viability\;\left(\%\right)=\frac{{OD}_{s}}{{OD}_{c}}\times 100$$ (6)

Where OD_s_ and OD_c_ are absorption of sample and control, respectively.

To investigate cell adhesion of samples, after 7 days of seeding cells on them, the media was extracted. The cells were fixed with glutaraldehyde (2.5% v/v) for 24 h. Then samples were dried with a series of ethanol solutions from 10 to 100% v/v and coated with gold to prepare for SEM analysis.

## Results and discussion

### Fourier transform spectroscopy analysis

**FIGURE 1:**
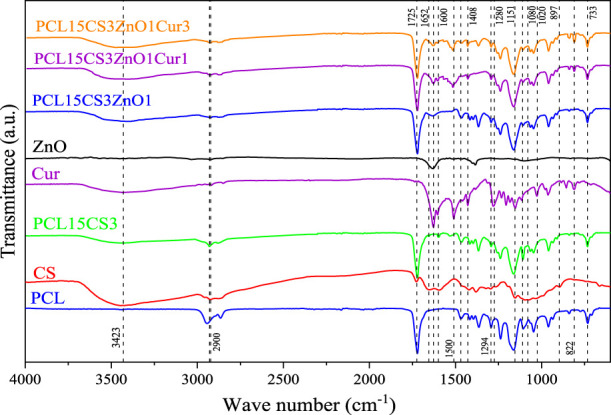
FTIR spectrum of PCL, CS, ZnO, Cur, and the electrospun samples.

The result of ATR-FTIR is shown in Figure 1 for pure PCL, CS, ZnO, Cur, and the electrospun nanofibers. Characteristic peaks of PCL include peaks at 733, 1294, 1469, and 1725 cm^−1^ which are attributed to C-O stretching, asymmetric stretching of C-O-C bridge, CH_2_ deformation, and carbonyl ester bonds (C=O), respectively (Ghaee et al., 2019). CS also showed its main characteristic peaks at 897, 1080, 1151, 1652 cm^−1^, 2922 cm^−1^, and 3423 cm^−1^, which belong to the CH_3_OH groups, C-O stretching, asymmetric stretching of C-O-C bridge, N-H bending, C-H stretching, and O-H stretching, respectively (Ghazalian et al., 2022). The characteristic peaks of PCL and CS exist in the FTIR of electrospun nanofiber of PCL15CS3. However, they shifted slightly due to the electrostatic interaction of PCL with CS. Also, the peaks at 2900 cm^−1^ and 1600 cm^−1^ belong to the stretching of C-H and bending of N-H, respectively. The peak at 1110 cm^−1^ is attributed to the C-O-C bridge.

The main characteristic peak of ZnO is located in the range of 500–700 cm^−1^ indicates the stretching of the Zn–O bond. The peak around 800 cm^−1^ can be attributed to the stretching vibration mode of Zn-O-Zn (Babaei et al., 2022). The main characteristic peaks of Cur are attributed to the aromatic peaks. Cur exhibits peaks at 822 cm^−1^ and 1020 cm^−1^ that are related to C-H aromatic in-plane bending modes and out-of-plane bending, respectively. The peaks at 1280, 1408,1510, and 1626 cm^−1^ belong to C–O–C stretching, aromatic C–O stretching, C=O stretching and C–C symmetric aromatic ring stretching, respectively (Ghaee et al., 2019). Characteristic peaks of ZnO and Cur are present with a shifted position in the spectrum of electrospun nanofibers containing them, confirming their presence.

### Scanning electron microscope images of samples

**FIGURE 2:**
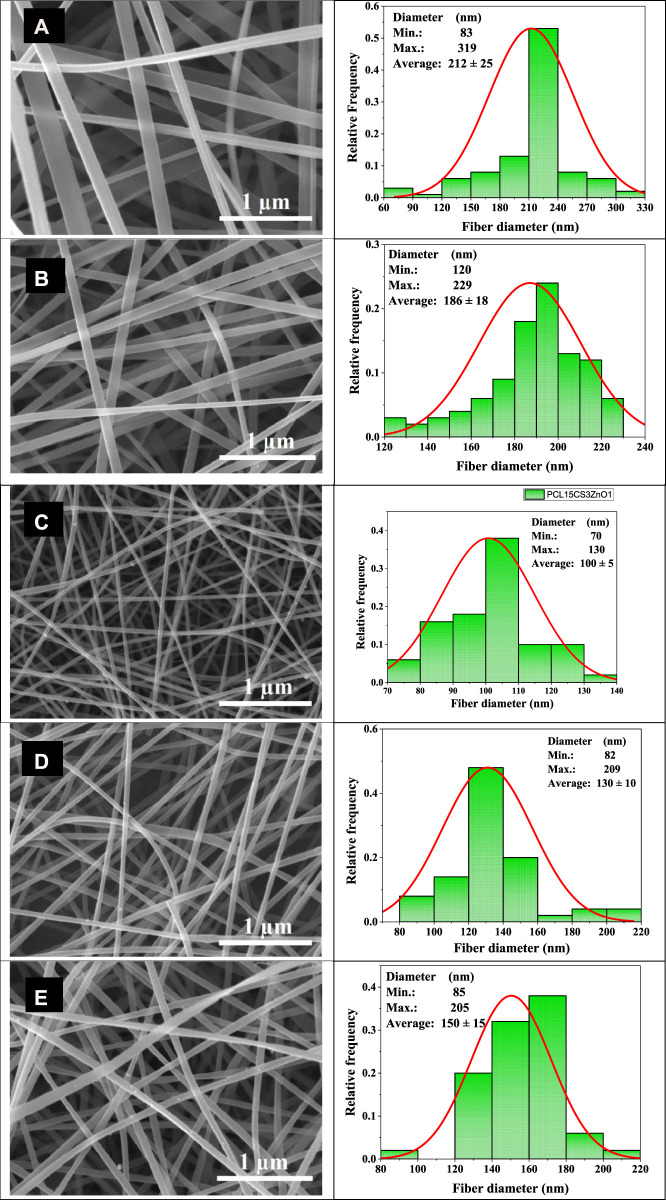
SEM images of the electrospun nanofibers and their diameter distribution histograms, **(A)** PCL15, **(B)** PCL15CS3, **(C)** PCL15CS3ZnO1, **(D)** PCL15CS3ZnO1Cur1, and **(E)** PCL15CS3ZnO1Cur3.

Figure 2 shows the SEM images of electrospun nanofibers and their diameter distribution histograms. All samples had uniform bead-free morphology. The pure PCL nanofibers showed smooth morphology with an average diameter of 212 ± 25 nm. The addition of CS decreased the diameter of nanofibers by about 186 ± 18 nm. The CS increases the polarity of electrospinning solution due to its charged functional groups that cause the stretching of fibers under an electrical field (Van der Schueren et al., 2012; Bolaina-Lorenzo et al., 2016). Moreover, ZnO nanoparticles increase conductivity of solution and cause more drawn. It can be seen that the PCL15CS3ZnO1 sample had the lowest average fiber diameter (100 ± 5 nm). The ZnO nanoparticles are also shown by the yellow arrow that have a good distribution state. The Cur drug affects viscosity more than conductivity. This increased solution viscosity led to increased average fiber diameters by 130 ± 10 and 150 ± 15 nm for nanofibers with 1 and 3 wt% Cur, respectively (Mitra et al., 2022).

### Elemental analysis of samples

**FIGURE 3:**
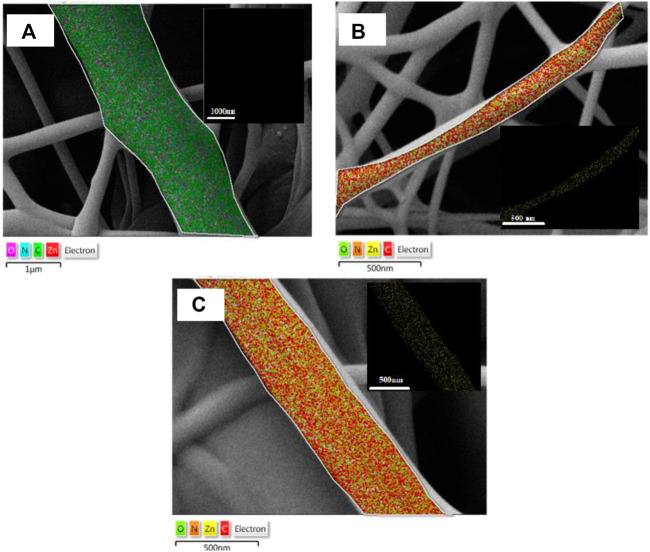
Elemental map of **(A)** PCL15CS3, **(B)** PCL15CS3ZnO1Cur1, and **(C)** PCL15CS3ZnO1Cur3.

The elemental maps of pure PCL15CS3 electrospun nanofibers and its composites containing ZnO and Cur are demonstrated in Figure 3. In these maps, zinc as the characteristic element of ZnO, and O, C, and N elements were detected. Based on this elemental map, ZnO nanoparticles (yellow points show Zn element) distributed homogeneously in samples of PCL15CS3ZnO1-Cur1 and PCL15CS3ZnO1Cur3. The obtained data from this analysis are also shown in Table 2.

**TABLE 2:** Data of the elemental analysis of the electrospun samples.

Sample	Carbon (C) atomic ratio (%)	Nitrogen (N) atomic ratio (%)	Oxygen (O) atomic ratio (%)	Zinc (Zn) atomic ratio (%)
PCL15CS3	69.8 ± 2.1	1.9 ± 0.8	28.3 ± 1.8	—
PCL15CS3ZnO1Cur1	69.3 ± 3.4	0.9 ± 0.6	29.1 ± 1.9	0.7 ± 0.4
PCL15CS3ZnO1Cur3	68.9 ± 3.1	0.8 ± 0.5	29.7 ± 1.6	0.6 ± 0.2

### X-ray diffraction analysis of samples

**FIGURE 4:**
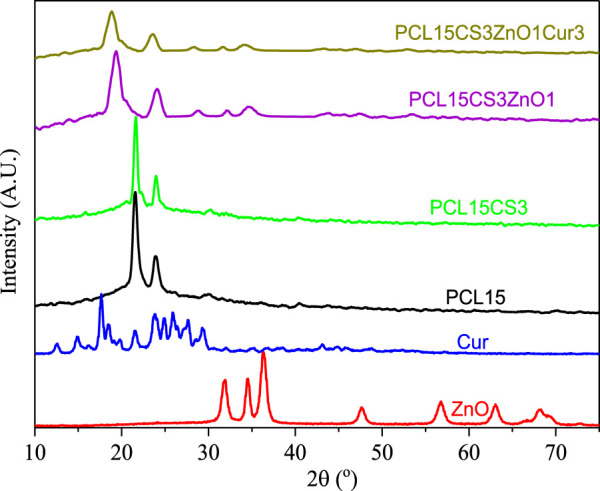
XRD pattern of Cur, ZnO, and the electrospun samples.

The XRD pattern of ZnO, Cur, and the electrospun nanofibers is shown in Figure 4. The X-ray diffractogram of ZnO showed several strong and sharp peaks at 2θ of 31.84°, 34.52°, 36.33°, 47.63°, 56.71°, and 68.13° which are related to planes of 100, 002, 101, 102, 110, and 112 and confirm the hexagonal structure of ZnO nanoparticles. The peak occurred at 34.52° is responsible the antibacterial capability of ZnO (Tajdari et al., 2021; Babaei et al., 2022). The exhibition of sharp peaks at 2θ of 17.3°, 21.3°, 23.4°, and 24.7° indicates high crystallinity of Cur (Rahimi et al., 2017).

The PCL15 nanofibers show two characteristic peaks at 2θ of 21.4° and 23.8° (Pekdemir et al., 2021). Although these two peaks appear in all electrospun nanofibers, they are slightly shifted to lower 2θ, which means PCL retained its crystallinity. By adding ZnO and Cur, the crystallinity of electrospun nanofibers was decreased due to the interaction of ZnO and Cur with the polymeric phase, which decreased the intensity of crystalline peaks in the PCL15CS3ZnO1Cur3 scaffold. The reduced mobility of polymer segments by CS and the blending of the elements in the amorphous phase can both contribute to the reduction of PCL crystallinity (Karthikeyan et al., 2020).

### Mechanical behavior of samples

**FIGURE 5:**
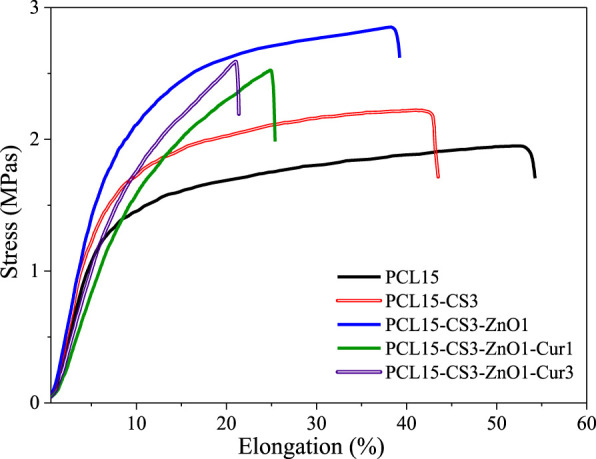
Stress-strain diagram of the electrospun nanofibers.

Electrospun nanofibers must possess appropriate mechanical properties especially elongation at break for wound dressing applications. It implies that a wound dressing must meet certain criteria to cover the wound without rupturing during the healing process. Figure 5 illustrates tensile stress-strain curves for electrospun nanofibers. The obtained results from the tensile test are summarized in Table 3. The tensile strength of electrospun nanofibers was between 1.9 ± 0.2 and 2.9 ± 0.5 MPa, which is in a good range for wound dressing application. Pure PCL15 nanofibers showed the lowest tensile strength and highest elongation at break (1.9 ± 0.2 MPa and 54 ± 3.2%, respectively). By blending PCL with CS, although the tensile strength increased to some extent (2.2 ± 0.3 MPa) compared to pure PCL15, but caused a decrease in elongation at break (43 ± 3.6%).

**TABLE 3:** Mechanical properties of the electrospun nanofibers.

Sample code	Tensile strength (MPa)	Tensile modulus (MPa)	Elongation at break (%)
PCL15	1.9 ± 0.2	0.25 ± 0.01	54 ± 3.2
PCL15CS3	2.2 ± 0.3	0.29 ± 0.02	43 ± 3.6
PCL15CS3ZnO1	2.9 ± 0.5	0.32 ± 0.04	39 ± 2.9
PCL15CS3ZnO1Cur1	2.5 ± 0.4	0.18 ± 0.03	25 ± 2.2
PCL15CS3ZnO1Cur3	2.6 ± 0.3	0.22 ± 0.02	22 ± 1.6

The high mechanical strength of ZnO as an inorganic phase and forming hydrogen bonds with the polymeric phase caused the highest tensile strength and modulus for PCL15CS3ZnO1 (2.9 ± 0.5 MPa and 0.32 ± 0.04 MPa) compared to other samples. The results also revealed that Cur weakened the mechanical properties of electrospun nanofibers in a dose-dependent manner. The tensile strength, tensile modulus, and elongation at break of the nanofibrous scaffold containing 1 and 3 wt% Cur were 2.5 ± 0.4 and 2.6 ± 0.3 MPa, 0.18 ± 0.03 and 0.22 ± 0.02 MPa and 25 ± 2.2 and 22 ± 1.6%, respectively.

### Thermogravimetric analysis analysis of samples

**FIGURE 6:**
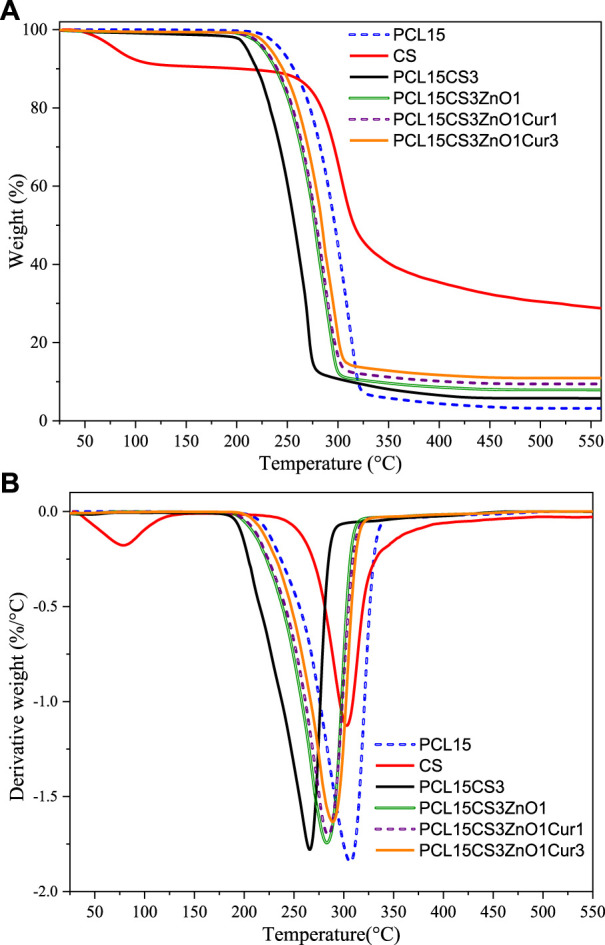
**(A)** TGA and **(B)** DTG curves of CS and electrospun samples.

The TGA curve of samples up to 550°C is shown in Figure 6. As can be seen, the thermogram of CS shows two-stage weight loss. The first decay happened at about 90°C due to the evaporation of intercalated or crystal moisture in the CS network, which was about 10% of the sample’s initial weight. In the second stage, about 60% of the weight of CS is lost, which is related to the breakdown of glycosidic bonds and the breakdown of CS chains consequently (Wanjun et al., 2005; Afshar et al., 2019). In addition, pure PCL15 has good thermal stability with the starting point of degradation about 230°C due to aliphatic polyester groups and its semi-crystalline structure (Pekdemir et al., 2021). By blending CS with PCL, the thermal stability of electrospun nanofibers decreased in comparison with pure CS and PCL15. The PCL15CS3 showed the lowest thermal stability and decomposition temperature (about 160–275°C) compared to other electrospun nanofibers.

The addition of ZnO shifted starting point of degradation to higher temperatures (from 160°C to about 200°C). The ZnO nanoparticles are inorganic compounds with high thermal conductivity that accelerates heat transfer to other parts of the electrospun nanofibers and delays degradation. The effect of 1 wt% Cur on the thermal degradation of electrospun nanofibers is negligible, but for 3 wt% Cur an enhancement is shown. Notably, thermogravimetric analysis of the nanofibers verified the lack of HFIP solvent in them (boiling point = 58.2°C), which guarantees the biocompatibility of electrospun nanofibers.

### Water contact angle

The contact angle between the water and the wound dressing provides useful information about the *in vivo* interaction between the surface of the damaged tissue and the wound dress. In general, increasing the hydrophilicity of the wound dressing surface leads to facilitated and improved cell adhesion and proliferation. The water contact angle (WCA) values of electrospun nanofibers are summarized in Table 4. The contact angle of PCL with water was about 118 ± 6.7° which is expected due to the hydrophobicity nature of PCL (Bolaina-Lorenzo et al., 2016). The addition of CS significantly increased the surface hydrophilicity of PCL nanofibers, which is thanks to the amino ester and hydroxyl groups in the CS chains (97 ± 5.1°). The ZnO also helped WCA decreases to 95 ± 4.5° because of increasing roughness and forming hydrogen bonds with water molecules. Although Cur does not dissolve in water, its oxygen can interact with water and serve as a proton donor. Therefore, the addition of Cur caused the nanofibers to become more hydrophilic and reduced the contact angle (Pulla Reddy et al., 1999; Nandhini et al., 2020).

**TABLE 4:** WCA values of the electrospun nanofibers.

Sample code	Contact angle (°)
PCL15	118 ± 6.7
PCL15CS3	97 ± 5.1
PCL15CS3ZnO1	95 ± 4.5
PCL15CS3ZnO1Cur1	89 ± 4.6
PCL15CS3ZnO1Cur3	78 ± 3.7

### Water uptake

**FIGURE 7:**
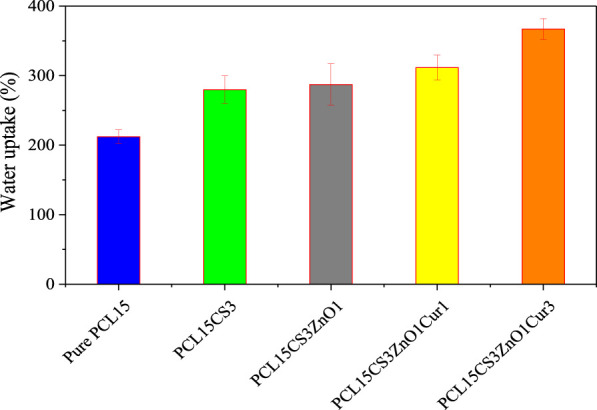
Water uptake of the electrospun samples.

The results of water uptake are summarized in Figure 7. To manage wound dryness and transfer nutrients and waste, optimum water uptake is necessary for wound dressing nanofibers. Water uptake of samples was about 210–370% which is appropriate for wound dressing. The pure PCL15 nanofibers had the lowest water uptake (212 ± 11%) compared to other samples, which is due to the hydrophobicity of t (Pekdemir et al., 2021). Notably, the water uptake capacity of a scaffold is influenced by several parameters such as including the polymer’s crystallinity, porosity, and hydrophilicity. The water uptake rises with increased amorphous area and porosity as a result of facilitated water penetration in micro and macro states. As can be seen, PCL15CS3 nanofibers had a greater water uptake compared to pure PCL15. This was caused by the hydrophilicity and amorphousness of the CS chains in comparison to PCL. This increases their affinity for water molecules and the porous structure of the CS-containing electrospun nanofibers, which allows water to be absorbed through the capillary effect. (Bolaina-Lorenzo et al., 2016; Fahimirad et al., 2021). ZnO nanoparticles increased water uptake of electrospun nanofibers by creating hydrogen bonds with water molecules. Although Cur has a hydrophobic nature, its hydrogen interactions with CS increase water uptake. The highest water uptake value (367 ± 15%) was achieved for PCL15CS3ZnO1Cur3.

### Water vapor transmission rate results

**FIGURE 8:**
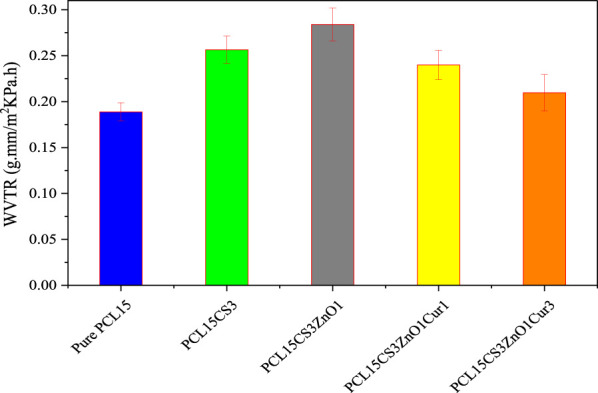
WVTR of the electrospun samples.

A dry wound or a wound infection can result from an imbalance of moisture and secretions between the wound dressing and the wound. To determine the gas exchange to the wound site, the WVTR of electrospun samples was investigated and summarized in Figure 8. According to the results, the presence of CS in the PCL15CS3 led to an increase in WVTR compared to pure PCL15 due to more hydrophilicity, amorphousness, and porosity. In WVTR, the porosity plays a more impressive role compared to hydrophilicity and amorphousness. Due to the increased scaffold porosity that resulted from a decrease in the nanofiber diameter (see Figure 2), WVTR has increased in the case of PCL15CS3ZnO1. Additionally, water vapor molecules and ZnO nanoparticles interact electrostatically, increasing water vapor permeability in contrast, in the case of nanofibers containing Cur, an increase in the nanofiber diameter results in a decrease in the scaffold porosity, which in turn causes a decrease in WVTR (Fahimirad et al., 2021). Besides, the WVTR was further reduced as Cur content was increased. Altogether, the results obtained from the fabricated nanofiber scaffolds proved appropriate water uptake and WVTR properties for application as a wound dressing.

### *In vitro* degradation

**FIGURE 9:**
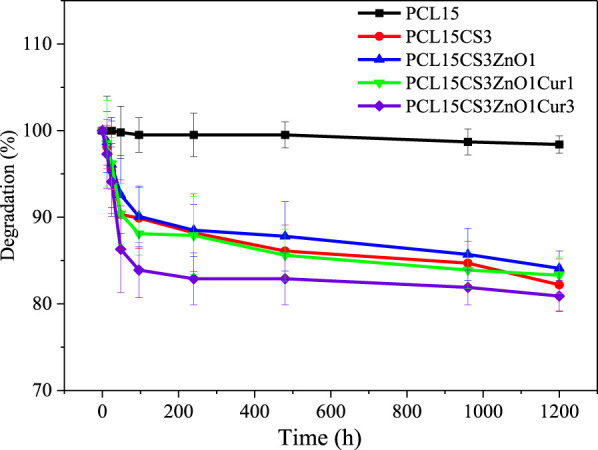
Degradation behavior of the electrospun samples.

Figure 9 shows the degradation behavior of electrospun nanofibers during 50 days. Based on the literature, the long linear aliphatic polyester structure of PCL slows down its degradation rate which is consistent with the results of the pure PCL15 sample (Sun et al., 2006). Due to the NH_2_ and OH groups that are found along the CS chains, which improve its hydrophilicity, CS can significantly contribute to boosting the degradation of electrospun nanofibers. In the other words, CS chains leak out of the scaffolds by dissolving in water over time (Sun et al., 2006). The effect of ZnO and 1 wt % Cur on the degradation of PCL15CS3 can be ignored, but PCL15CS3ZnO1Cur3 had the highest degradation.

### *In vitro* release of Cur

**FIGURE 10:**
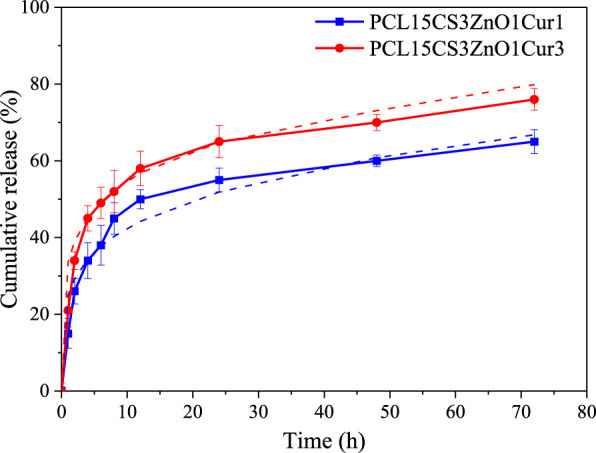
Release profile with their fitted Peppas model of the Cur-containing electrospun samples.

Figure 10 illustrates the cumulative release profile of Cur drug in electrospun nanofibers with 1 and 3 wt% Cur. According to the results, both nanofibers present a two-stage Cur release, a burst release followed by a steady one. The burst release of PCL15CS3ZnO1Cur3 (34 ± 2.3 and 52 ± 5.5% of Cur in the first 2 and 6 h) is more than PCL15CS3ZnO1Cur1 (26 ± 3.3 and 45 ± 5.2% of Cur in the first 2 and 6 h) due to higher Cur content. The release of Cur at the beginning of the steady release stage (during 24 h) was 65 ± 4.2 and 55 ± 3.1% for PCL15CS3ZnO1Cur3 and PCL15CS3ZnO1Cur1, respectively. After 72 h, electrospun nanofibers of PCL15CS3ZnO1Cur3 and PCL15CS3ZnO1Cur1 released 76 ± 2.8 and 65 ± 3.1% of Cur, respectively.

Finally, six models were used to analyze the kinetics of drug release, which are listed in the Supplementary Material (Supplementary Table S2). The results of fitting for each model are summarized in Table 5. Among the presented models, the Peppas one had the highest correlation coefficient (R^2^) and the best fitting (See Figure 10). The plots for other curve-fitting analysis are displayed in the Supplementary Material (Supplementary Figures S1–S5). Based on the Peppas model, the exponent (n) is used for describing the release mechanisms. *n* = 0.5 characterizes typical Fickian diffusion, whereas *n* = 1 describes case II diffusion. A non-Fickian is indicated by n values between 0.5 and 1. Predicted n exponents for both samples are below 0.5 which reveals the mechanism of drug release from electrospun nanofibers is non-Fickian diffusion (Shahrousvand et al., 2022). The correlation coefficients of the model for two Cur-containing samples was 0.93 and 0.95, indicating that the model fits the experimental data reasonably well.

**TABLE 5:** Parameters of different kinetic models for the release profile of Cur-containing electrospun samples.

Kinetic models	PCL15CS3ZnO1Cur1	PCL15CS3ZnO1Cur3
Zero order	$K_{0}^{\prime \prime }$ = 0.005, R^2^ = 0.669	$K_{0}^{\prime \prime }$ = 0.006, R^2^ = 0.672
First order	K_1_ = 0.013, R^2^ = 0.490	K_1_ = 0.011, R^2^ = 0.499
Higuchi	K'_H_ = 0.058, R^2^ = 0.836	K'_H_ = 0.062, R^2^ = 0.835
Hixson-Crowell	K_HC_ = 0.003, R^2^ = 0.743	K_HC_ = 0.003, R^2^ = 0.777
Baker-Lonsdale	K_BL_ = 0.001, R^2^ = 0.859	K_BL_ = 0.002, R^2^ = 0.881
Peppas	K = 24.97, R^2^ = 0.952, *n* = 0.23	K = 32.75, R^2^ = 0.934, *n* = 0.21

### Antibacterial efficiency of samples

**FIGURE 11:**
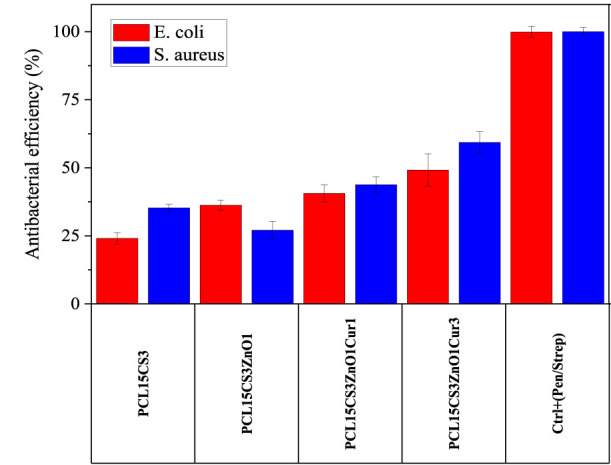
Antibacterial efficiency of the electrospun samples against *E. coli* and *S. aureus* bacteria after 24 h.

The antibacterial activity of electrospun nanofibers against two types of bacteria, *Escherichia coli* (*E. coli*) and *Staphylococcus aureus* (*S. aureus*), was investigated (Figure 11). As expected, pure PCL15 did not show any inhibition. The results showed that the incorporation of CS into the PCL scaffold was effective in increasing the antibacterial efficiency against both bacteria. The PCL15CS3 scaffold had an antibacterial efficiency of 24.1 ± 2.1 and 35.3 ± 1.3 against *E. coli* and *S. aureus*, respectively. This is due to the interaction of NH_2_ groups of CS with PCL chains. Moreover, CS is an effective natural antibacterial agent against both bacteria and chitosan’s positive charge interacts with the negatively charged cell membranes to kill bacteria (Ono et al., 2000; Zou et al., 2020).

ZnO nanoparticles had different effects on the antibacterial activity of the scaffolds against bacteria, increasing antibacterial activity against *E. coli* while decreasing antibacterial effectiveness against *S. aureus*. It means that ZnO had a better performance against *E. coli* bacteria, which is due to their thin-walled nature (Bakhsheshi-Rad et al., 2021; Keyvani et al., 2021). Incorporation of Cur to PCL15CS3ZnO1 produced greater antibacterial activity against both bacteria. For example, PCL15CS3ZnO1Cur3 provided an antibacterial efficiency of 49.2 ± 5.8 and 59.3 ± 3.9% against *E. coli* and *S. aureus* bacteria, respectively. These findings demonstrated that all nanofibers had appropriate antibacterial activity, making them viable scaffolds for use in wound dressings.

### MMT and cell adhesion assay

**FIGURE 12:**
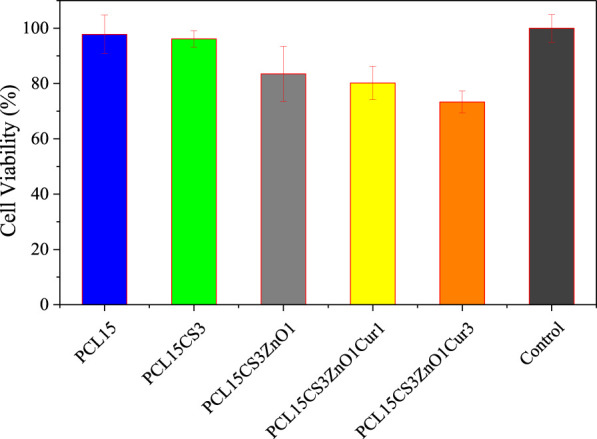
Cell viability of the electrospun samples after 24 h.

In order to study the cell viability of electrospun scaffolds, L929 cells were cultured directly on the nanofibers for 24 h. The cell viability of electrospun nanofibers is shown in Figure 12. In the case of Cur-free samples, cell viability was greater than 80%, demonstrating that there was no cytotoxicity. However, cell viability is decreased by the addition of ZnO and Cur. In other words, Cur inhibited the proliferation and survival of L929 cells in a dose-dependent manner.

**FIGURE 13:**
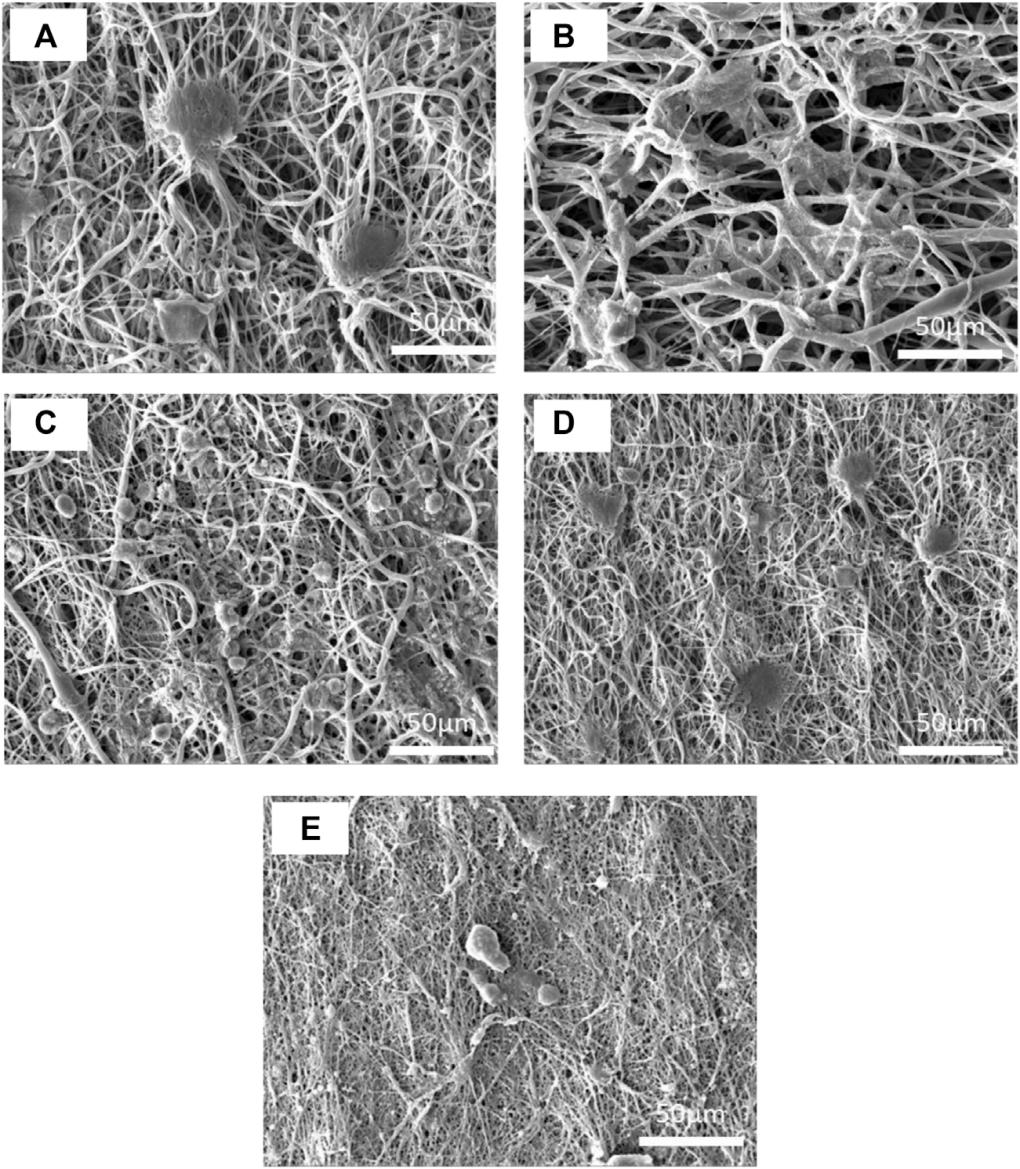
SEM images of L 929 fibroblast cells on the electrospun nanofibers of **(A)** PCL15, **(B)** PCL15CS3, **(C)** PCL15CS3ZnO1, **(D)** PCL15CS3ZnO1Cur1, and **(E)** PCL15CS3ZnO1Cur3 after 7 days cell seeding.

The cell morphology on the electrospun nanofibers is illustrated in Figure 13. Despite the pure PCL15 sample having high cell adhesion, there were undesirable cell distributions on the electrospun mat. The increase in cell adhesion on PCL15CS3 nanofibers is due to the polysaccharide structure of CS and its higher hydrophilicity compared to PCL. Cell shrinking and the spherical phenotype of cells are early signs of apoptosis. According to this phenomenon, ZnO nanoparticles and Cur decreased cell adhesion and cell distribution which is in agreement with MTT results.

## Conclusion

In this study, the PCL and CS mixture was successfully electrospun using a 15 wt% PCL and a 3 wt% CS in HFIP solvent to improve the hydrophilicity of PCL nanofibers for use in wound dressing applications. Afterward, Cur and ZnO were incorporated into the electrospun nanofibers, and their structural, physicomechanical, antibacterial activity, and *in vitro* properties were investigated. SEM analysis showed smooth nanofibers with a bead-free morphology. The elemental analysis also proved a good distribution of ZnO in scaffolds. By adding ZnO, WVTR and nanofibers’ tensile characteristics were significantly improved, while the highest water uptake value was achieved for the sample containing 3 wt% Cur. The Cur drug released rapidly and reached a steady state about 24 h. The Peppas model provided the best fitting results on experimental data. Simultaneous incorporation of Cs, ZnO, and Cur effectively inhibited bacterial growth. The outcomes showed that electrospun nanofibers made of PCL, CS, ZnO, and Cur had a high potential for use as wound dressings.

## Data Availability

The original contributions presented in the study are included in the article/Supplementary Material, further inquiries can be directed to the corresponding author.
