# Supplementary material for: Fabrication and characterization of polycaprolactone/chitosan nanofibers containing antibacterial agents of curcumin and ZnO nanoparticles for use as wound dressing
Source: Front Bioeng Biotechnol. 2022 Sep 23;10:1027351. doi: 10.3389/fbioe.2022.1027351 (PMC9539460; doi:10.3389/fbioe.2022.1027351)
Supplement: Supplementary file 1 [file DataSheet1.pdf]

Supplementary Material

Fabrication and Characterization of Polycaprolactone / Chitosan Nanofibers Containing Antibacterial Agents of Curcumin and ZnO Nanoparticles for Use as Wound Dressing

Pezhman Mosallanezhad[1], Hossein Nazockdast[2*], Zahed Ahmadi[3], Amir Rostami[4]

[1] Department of Polymer Engineering, Amirkabir University of Technology, Mahshahr, Iran. Email: pejman.mosallanejad@gmail.com.

[2] Department of Polymer Engineering and Color Technology, Amirkabir University of Technology, Tehran, Iran. Email: nazdast@aut.ac.ir.

[3] Department of Chemistry, Amirkabir University of Technology, Tehran, Iran. Email: zahmadi@aut.ac.ir.

[4] Department of Chemical Engineering, Faculty of Petroleum, Gas, and Petrochemical Engineering, Persian Gulf University, Bushehr, 75169-13817, Iran. Email: arostami@pgu.ac.ir.

*** Correspondence:**
Prof. Hossein Nazockdast
E-mail address: nazdast@aut.ac.ir.

The Supplementary material has 9 pages and it contains 2 Tables and 5 Figures.

The results of a full factorial design experiment to optimize the electrospinning conditions are shown in this supplementary material. The PCL and CS concentrations, flow rate, voltage, needle-to-collector distance, and collector speed are the electrospinning parameters that are taken into consideration. Despite the fact that needle-to-collector distance and collector speed only have a small impact, they were both fixed at 15 cm and 300 rpm, respectively.

As can be seen from Table S1, a complete factorial design model was developed, having four controllable factors with two levels (PCL concentration of 10 and 15 wt%, CS concentration of 3 and 5 wt%, flow rate of 0.5 and 1 $mL.h^{-1}$, and applied voltage of 10 and 15 kV). The objective is to identify the best conditions that produce a desired nanofibrous scaffold with smooth surfaces, bead-free morphology, and uniform diameter distribution (1). With these all, according to SEM images, the S8 sample was selected as the best one among others.

Table S1. SEM images of the electrospun scaffolds with different PCL and CS concentrations, flow rates, and voltages. All scale bars are 1 μm.

Run	PCL Conc. (wt%)	CS Conc. (wt%)	Voltage (kV)	Flow rate (mL/h)	SEM Image
S1	10	3	10	0.5	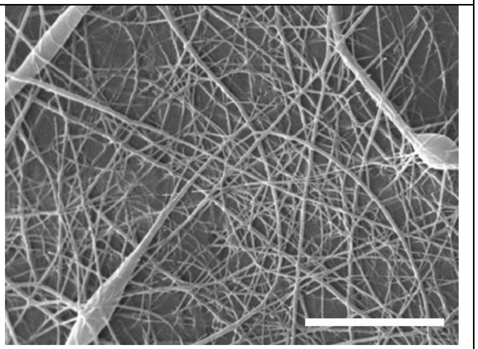
S2	10	3	15	0.5	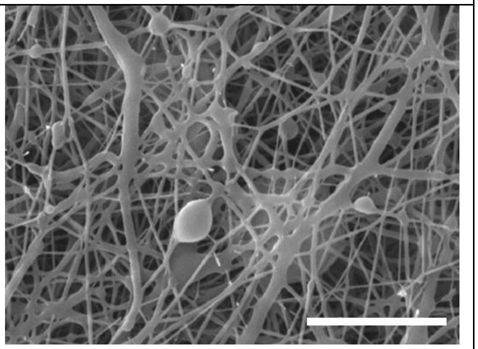

S3	10	3	10	1	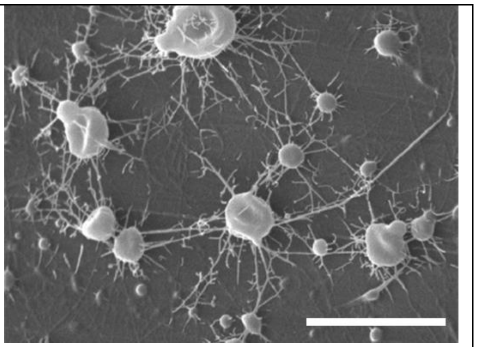
S4	10	3	15	1	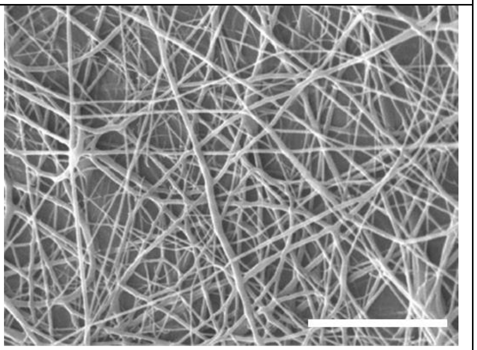
S5	15	3	10	0.5	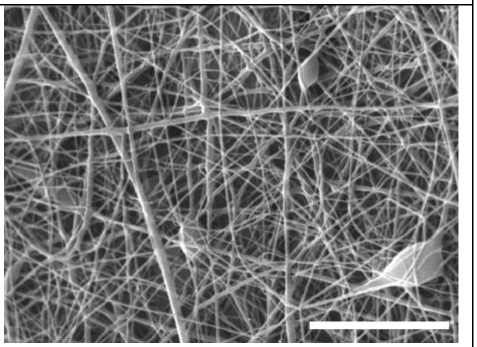
S6	15	3	15	0.5	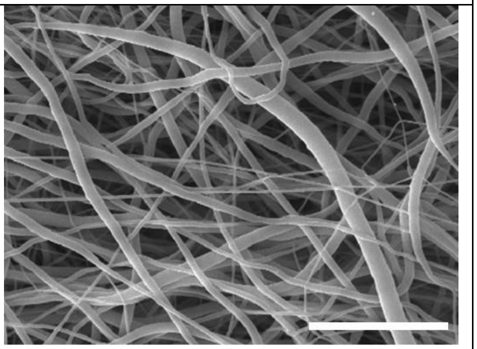

S7	15	3	10	1	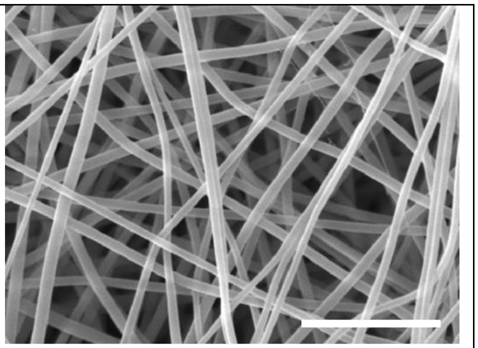
S8	15	3	15	1	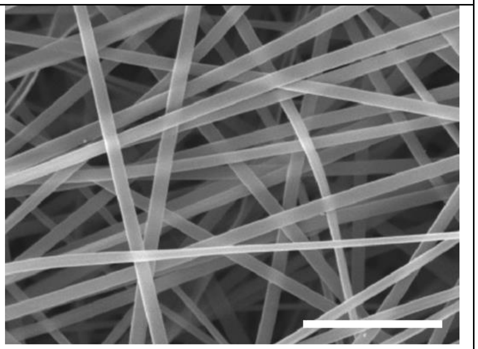
S9	10	5	10	0.5	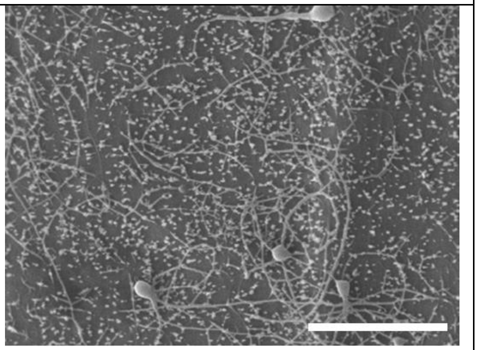
S10	10	5	15	0.5	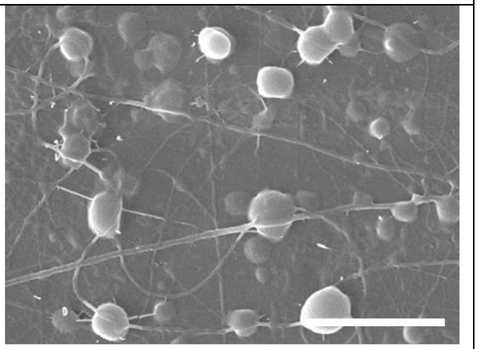

S11	10	5	10	1	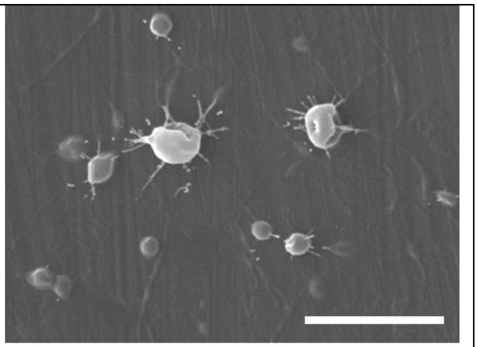
S12	10	5	15	1	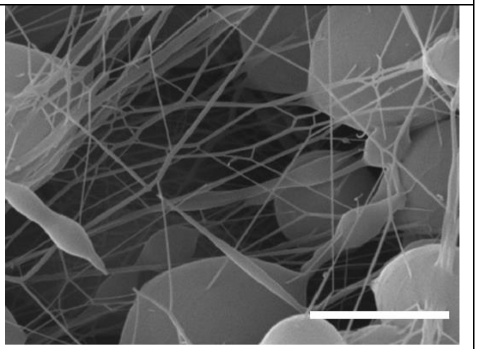
S13	15	5	10	0.5	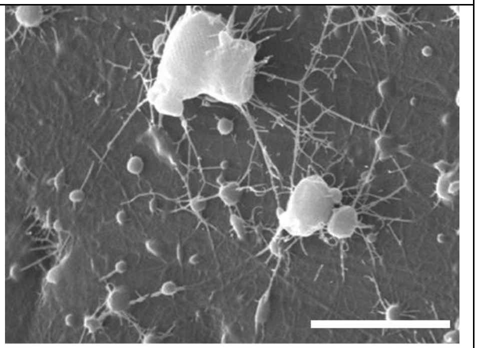
S14	15	5	15	0.5	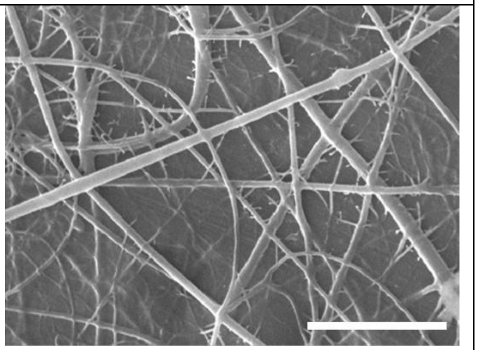

S15	15	5	10	1	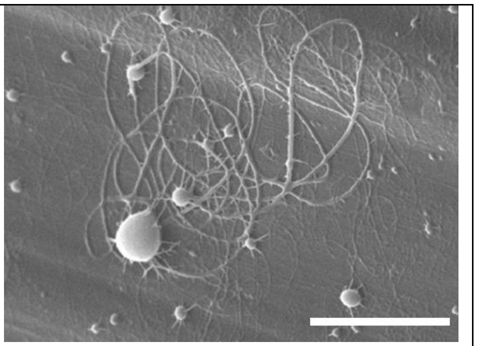
S16	15	5	15	1	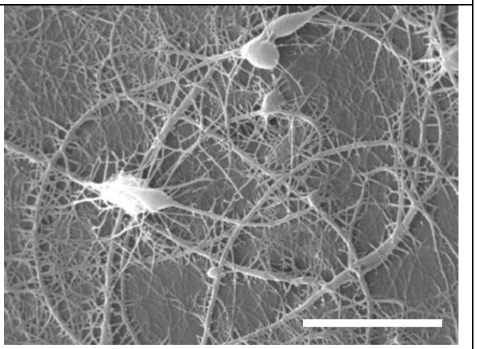

Table S2 lists the models that were used to analyze the kinetics of drug release.

Table S2. Utilized kinetic models for tracking the drug release mechanism (2).

Kinetic models	Formulation	Details
Zero order	$Q_t = \frac{M_t}{M_\infty} = K_0''t$	K_0'' = The zero order constant<br>Q_t: The fraction of released drug in the time interval of t.<br>M_t and M_∞ are the amount of drug released at time *t* and the total amount of drug, respectively.
First order	$\log Q_t = \log Q_0 + \frac{K_1 t}{2.303}$	Q_0: is initial amount of drug dissolved.<br>K_1: The first order constant.
Higuchi	$Q_t = K'_H\sqrt{t}$	$K'_H = \frac{K_H}{M_\infty}$<br>$K_H$: The Higuchi constant.
Hixson-Crowell	$\sqrt[3]{1 - Q_t} = 1 - K_\beta t$	$K_\beta = \frac{K_{HC}}{\sqrt{M_0}}$<br>$K_{HC}$: The Hixson-Crowell constant
Baker-Lonsdale	$\frac{3}{2}\left(1 - (1 - Q_t)^{2/3}\right) - Q_t = K_{BL}t$	Q_t: The fraction of released drug in the time interval of t.<br>K_{BL}: The Baker-Lonsdale constant.
Peppas	$Q_t = Kt^n$	K: Peppas constant.<br>n: The Peppas exponent.

Figures S1 to S5 display the plots for curve-fitting analysis.

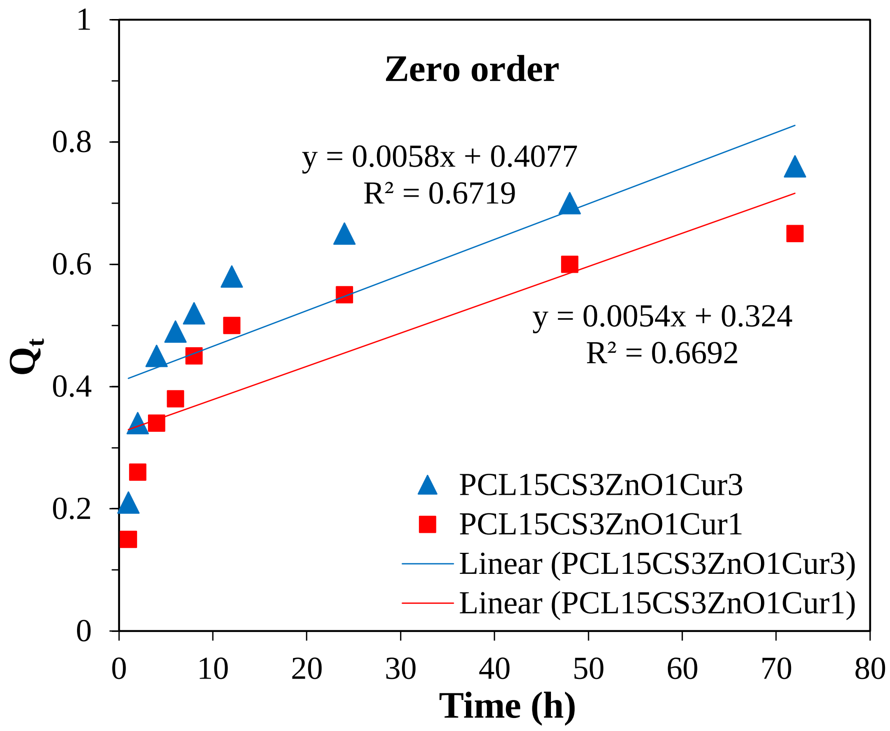

Figure S1. Kinetic information of Cur release and fitting with Zero order model.

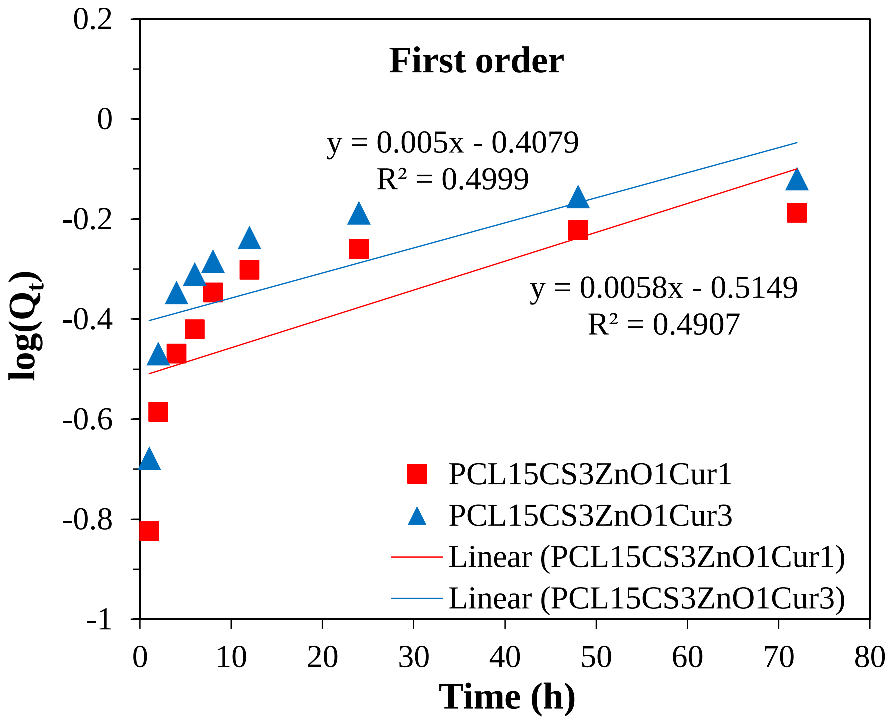

Figure S2. Kinetic information of Cur release and fitting with First order model.

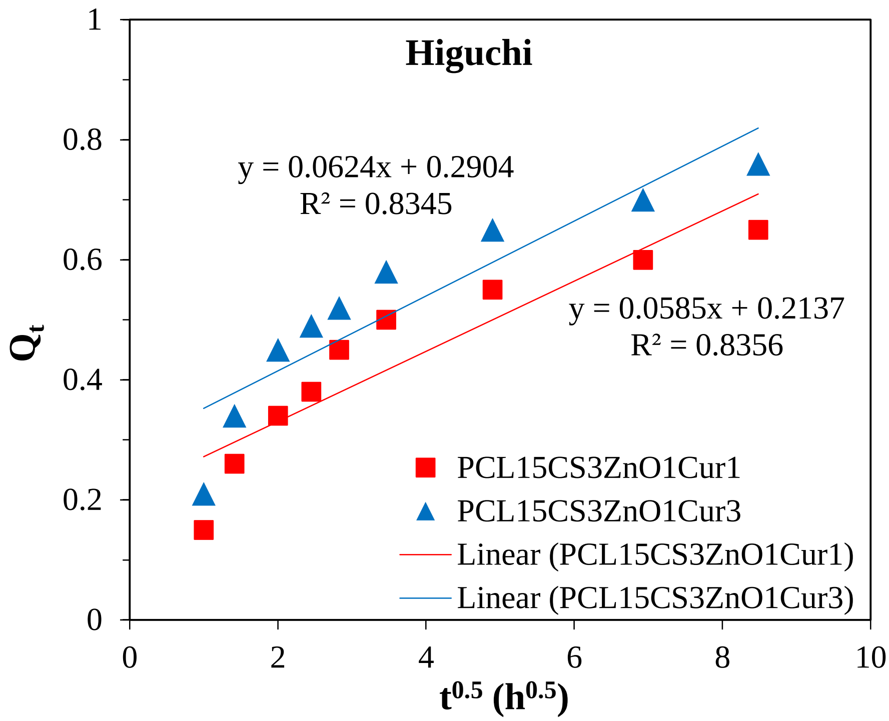

Figure S3. Kinetic information of Cur release and fitting with Higuchi model.

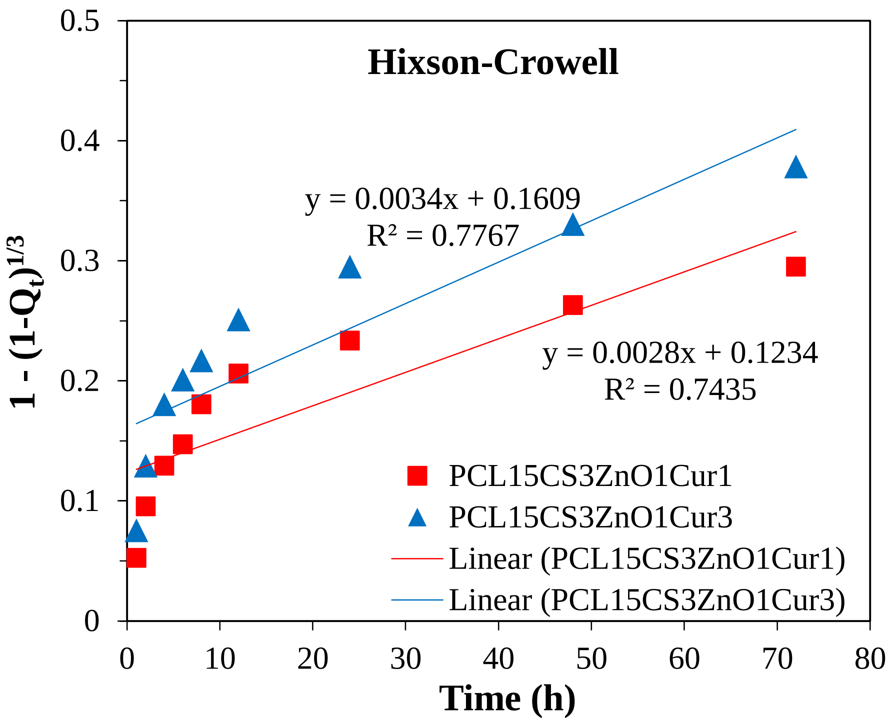

Figure S4. Kinetic information of Cur release and fitting with Hixson-Crowell model.

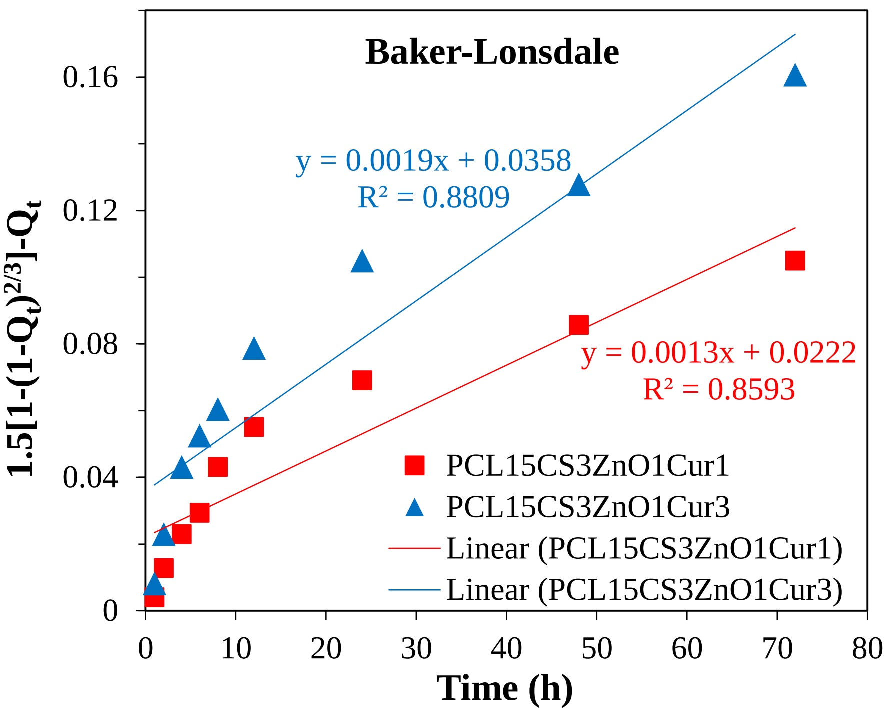

Figure S5. Kinetic information of Cur release and fitting with Baker-Lonsdale model.

References:

1. Ghazalian M, Afshar S, Rostami A, Rashedi S, Bahrami SH. Fabrication and characterization of chitosan-polycaprolactone core-shell nanofibers containing tetracycline hydrochloride. Colloids and Surfaces A: Physicochemical and Engineering Aspects. 2022;6.36.128163.
2. Ambekar RS, Kandasubramanian BJEPJ. Advancements in nanofibers for wound dressing: A review. 2019;117:304-36.
